# Ω3 Supplementation and Intermittent Hypobaric Hypoxia Induce Cardioprotection Enhancing Antioxidant Mechanisms in Adult Rats

**DOI:** 10.3390/md13020838

**Published:** 2015-02-04

**Authors:** Emilio A. Herrera, Jorge G. Farías, Alejandro González-Candia, Stefania E. Short, Catalina Carrasco-Pozo, Rodrigo L. Castillo

**Affiliations:** 1 Programa de Fisiopatología, Instituto de Ciencias Biomédicas, Facultad de Medicina, Universidad de Chile, Santiago 8380453, Chile; E-Mails: eherrera@med.uchile.cl (E.A.H.); alejjobq@gmail.com (A.G.-C.); 2 Departamento de Ingeniería Química, Facultad de Ingeniería y Ciencias, Universidad de la Frontera, Temuco 4811230, Chile; E-Mails: Jorge.farias@ufrontera.cl (J.G.F.); s.short01@ufromail.cl (S.E.S.); 3 Departamento de Nutrición, Facultad de Medicina, Universidad de Chile, Santiago 8380453, Chile; E-Mail: catalinacarrasco@med.uchile.cl

**Keywords:** intermittent hypobaric hypoxia, high-altitude, oxidative stress, inflammation, ventricular function, cardiac impairment, ischemia-reperfusion, Ω3 fatty acids

## Abstract

Intermittent hypobaric hypoxia (IH) is linked with oxidative stress, impairing cardiac function. However, early IH also activate cardio-protective mechanisms. Omega 3 fatty acids (Ω3) induce cardioprotection by reducing infarct size and reinforcing antioxidant defenses. The aim of this work was to determine the combined effects of IH and Ω3 on cardiac function; oxidative balance and inflammatory state. Twenty-eight rats were randomly divided into four groups: normobaric normoxia (N); N + Ω3 (0.3 g·kg^−1^·day^−1^); IH; and IH + Ω3. IH was induced by 4 intercalate periods of hypoxia (4 days)—normoxia (4 days) in a hypobaric chamber during 32 days. At the end of the exposure, hearts were mounted in a Langendorff system and subjected to 30 min of ischemia followed by 120 min of reperfusion. In addition, we determined HIF-1α and ATP levels, as well as oxidative stress by malondialdehyde and nitrotyrosine quantification. Further, the expression of the antioxidant enzymes superoxide dismutase, catalase, and glutathione peroxidase was determined. NF-kappaB and myeloperoxidase levels were assessed in the hearts. Relative to N hearts, IH improved left ventricular function (Left ventricular developed pressure: N; 21.8 ± 3.4 *vs.* IH; 42.8 ± 7.1 mmHg; *p* < 0.05); reduced oxidative stress (Malondialdehyde: N; 14.4 ± 1.8 *vs.* IH; 7.3 ± 2.1 μmol/mg prot.; *p* < 0.05); and increased antioxidant enzymes expression. Supplementation with Ω3 induces similar responses as IH group. Our findings suggest that both, IH and Ω3 in an independent manner, induce functional improvement by antioxidant and anti-inflammatory mechanisms, establishing cardio-protection.

## 1. Introduction

Intermittent hypoxia (IH) is experienced by a great number of Andean workers (*i.e*., minery, astronomy observatory, customs and boundary armed forces), being a unique model of exposure to hypoxia whereby periods of stay at higher altitude are interspersed with periods of stay at sea level. These periods may be as short as one day to several days [1,2], and are a different cardiovascular challenge compared to acute (sport and tourism), episodic (sleep apnea) or chronic (permanent residence) exposure. The response to episodic and chronic hypoxia is characterized by marked cardiovascular effects to offset a global decrease in tissue oxygen supply, including polycythemia and an overall sympathetic stimulation. Similarly to sustained hypoxia, there is a hypoxic pulmonary vasoconstriction, which leads to pulmonary hypertension and right ventricular hypertrophy if the shift exposure is prolong [3]. Moreover, IH is associated with the development of systemic hypertension and left ventricular dysfunction [4]. At present, however, our understanding of the basic mechanisms linking IH and cardiovascular dysfunction is limited by the pathophysiological heterogeneity of hypoxic patients and the presence of multiple confound and comorbid conditions, including obesity and previous cardiac impairments [5]. Moreover, the great variety of responses range from no clinical effects to strong pulmonary hypertension and vital risk. Consequently, there is a serious need for the development of experimental models to study the mechanisms involved in the cardiovascular responses to IH and the potential deleterious effects [6].

At physiologic concentrations, reactive oxygen species (ROS) are involved in the adaptive responses to hypoxia (both acute and intermittent), given their capacity as signaling molecules to activate oxygen-sensitive genes [7]. In excess, however, ROS are equally capable of causing structural cell-membrane damage, vascular endothelial dysfunction, and promoting cardiovascular diseases [8]. Accordingly, acute exposure to high altitude has been shown to induce oxidative stress in healthy human lowlanders, as indicated by an increase in free radical formation [9,10]. Several sources of ROS are activated during exposure to high altitude, including the mitochondrial electron transport chain [11], xanthine oxidase, NADPH oxidase and nitric oxide synthase [12]. Moreover, the enzymatic and non-enzymatic antioxidant systems are potentially depleted by exposure to high altitude, such as glutathione peroxidase, superoxide dismutase activities and total antioxidant capacity [13,14]. Specifically in the heart, chronic IH may induce left ventricular oxidative damage and hypertrophy, impairing the contractile capacity [15,16,17]. However, IH may induce a ischemic preconditioning-like effect in the heart as well [18,19]. The protective effects promote recovery of cardiac contractile function from an ischemic-reperfused (IR) condition, limiting infarct size and decreasing reperfusion arrhythmias [20,21]. Several mechanisms have been proposed to be involved in the protective mechanism afforded by chronic IH, including regulation of myocardial heat shock protein expression, amelioration of coronary circulation and angiogenesis, activation of protein kinase C and involvement of K_ATP_ channels activity [22,23]. However, the complete effects and involved mechanisms of chronic IH on cardiac function are still unknown. 

Compelling data show cardiovascular beneficial effects in consuming fatty acids highly present in fish, such as omega 3 (Ω3), docosahexanoic acid (DHA 22:6 Ω3), and eicosapentanoic acid (EPA 20:5 Ω3). These fatty acids regulate cell membrane physicochemical properties (*i.e.*, fluidity, organization and permeability) that affect signaling pathways and diffusion processes, with positive effects on key cardiovascular pathways [24]. Additionally, Ω3 regulate the synthesis of immune system mediators (*i.e.*, thromboxanes, prostacyclins, prostaglandins, leukotrienes, hydroeicosatetranoic acids, and lipoxins) altering the arachidonic acid metabolism and reducing pro-inflammatory responses [25,26]. In addition, Ω3 can improve post-ischemic functional recovery in isolated rat hearts, suggesting the benefits of highly enriched Ω3 content diet [27]. In fact, regular intake can slow the heart rate, reduce myocardial oxygen consumption, and increase coronary reserve [28]. These properties probably contribute to protective preconditioning-like effects on the myocardial IR damage and improve post-ischemic recovery.

Therefore, we hypothesize that chronic IH induces preconditioning-like responses in short-term cycles mimicking the ischemia-reperfusion shifts, and that these outcomes could be enhanced by the Ω3 cardioprotective effects. The aim of this work was to determine the combined effects of IH and Ω3 on cardiac function, oxidative tone and inflammatory status.

## 2. Results

### 2.1. Body Weight and Cardiac Parameters

All groups increased body weight during the exposition period. Previous euthanasia, N rats showed a body weight of 326 ± 35 g. Further, N + Ω3 group showed 21.8% (442 ± 42 g) increased weight relative to N (*p* < 0.05). In contrast, IH rats markedly decreased body weight by 18.9% (265 ± 38 g) relative to N (*p* < 0.05). This hypoxia-induced effect was fully reverted in the IH + Ω3 (308 ± 25 g). Conversely, IH rats showed increased values of energy consumption (28.8 ± 1.8 Kcal 100 g body weight/day, *p* < 0.05) relative to N (20.8 ± 2.5 Kcal 100 g body weight/day). Further, energy consumption N + Ω3 and IH + Ω3 were similar (21.9 ± 2.4 and 22.6 ± 3.3 Kcal 100 g body weight/day, respectively), showing that Ω3 reverses hypoxia-induced energy consumption. Further, the heart-to-body weight ratio and heart rate showed non-significant differences between groups (Table 1). 

**Table 1:** Cardiac parameters at the end of the exposure protocol.

	N	N + Ω3	IH	IH + Ω3
	(*n* = 7)	(*n* = 7)	(*n* = 7)	(*n* = 7)
**Cardiac/Body Weight**				
mg/g	0.31 ± 0.09	0.33 ± 0.10	0.36 ± 0.15	0.28 ± 0.07
**Heart Rate**				
beats/min	310 ± 2.2	295 ± 1.8	308 ± 1.7	298 ± 2.3

Groups are normobaric normoxic (N), N + omega 3 (N + Ω3), intermittent hypobaric hypoxic (IH) and IH + omega 3 (HI + Ω3). Data expressed as mean ± SEM.

### 2.2. Left Ventricular Function

**Figure 1:**
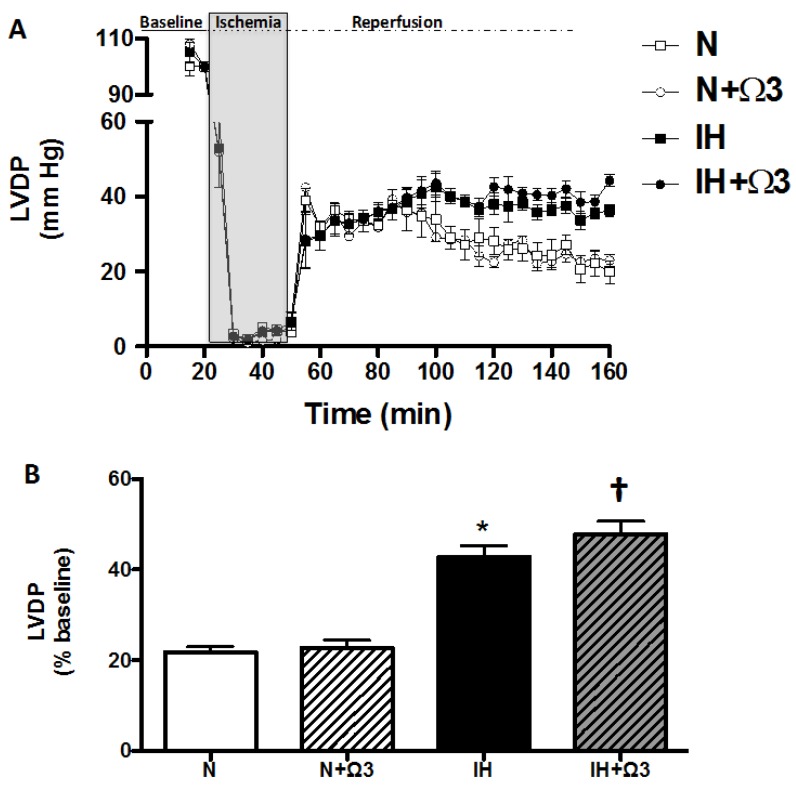
*Left ventricular developed pressure (LVDP).* Continuous measurement of LVDP along the Langendorff experimental protocol (**A**) and at average at 120 min of reperfusion (**B**). Groups are normobaric normoxic (N), N + omega 3 (N + Ω3), intermittent hypobaric hypoxic (IH) and IH + omega 3 (IH + Ω3). Data expressed as mean ± SEM (*n* = 7 per group). Significant differences (*p* < 0.05): * *vs.* N; **^†^** *vs.* IH.

At baseline, LVDP and LVEDP were similar between the four experimental groups. However, at 120 min of reperfusion after ischemia, hearts of IH and IH + Ω3 groups exhibited significant increases in LVDP (Figure 1) and decreases in LVEDP relative to N and N + Ω3 groups (Table 2) (*p* < 0.05).

**Table 2:** Left ventricular end diastolic pressure at baseline and after ischemia-reperfusion.

	N	N + Ω3	IH	IH + Ω3
	(*n* = 7)	(*n* = 7)	(*n* = 7)	(*n* = 7)
**Baseline values LVEDP (mmHg)**	5.7 ± 0.8	5.1 ± 0.5	6.1 ± 0.9	5.5 ± 0.7
**After 120 min reperfusion LVEDP (mmHg)**	34.9 ± 1.8	33.1 ± 2.3	21.7 ± 1.9 *	20.6 ± 3.7 *

Groups are normobaric normoxic (N), N + omega 3 (N + Ω3), intermittent hypobaric hypoxic (IH) and IH + omega 3 (HI + Ω3). Data expressed as mean ± SEM. Significant differences (*p* < 0.05): * *vs.* N or N **+ **Ω3 (as appropriate).

### 2.3. LDH Levels

**Figure 2:**
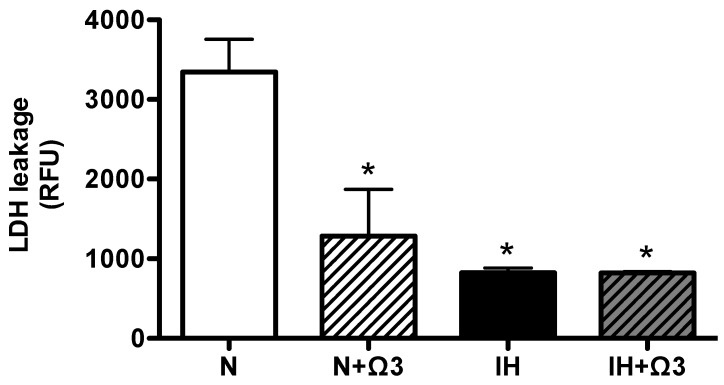
*Lactate dehydrogenase in plasma (LDH).* LDH leakage in plasma samples of rats at the end of protocol exposure protocol. Groups are normobaric normoxic (N), N + omega 3 (N + Ω3), intermittent hypobaric hypoxic (IH) and IH+omega 3 (IH + Ω3). Data expressed as mean ± SEM (*n* = 7 per group). Significant differences (*p* < 0.05): * *vs.* N or N + Ω3 (as appropriate).

As an index of cell death we measured plasmatic LDH. Interestingly, LDH was markedly reduced in N + Ω3, IH and IH + Ω3, in similar magnitude relative to N (Figure 2).

### 2.4. HIF-1α and ATP Levels

**Figure 3:**
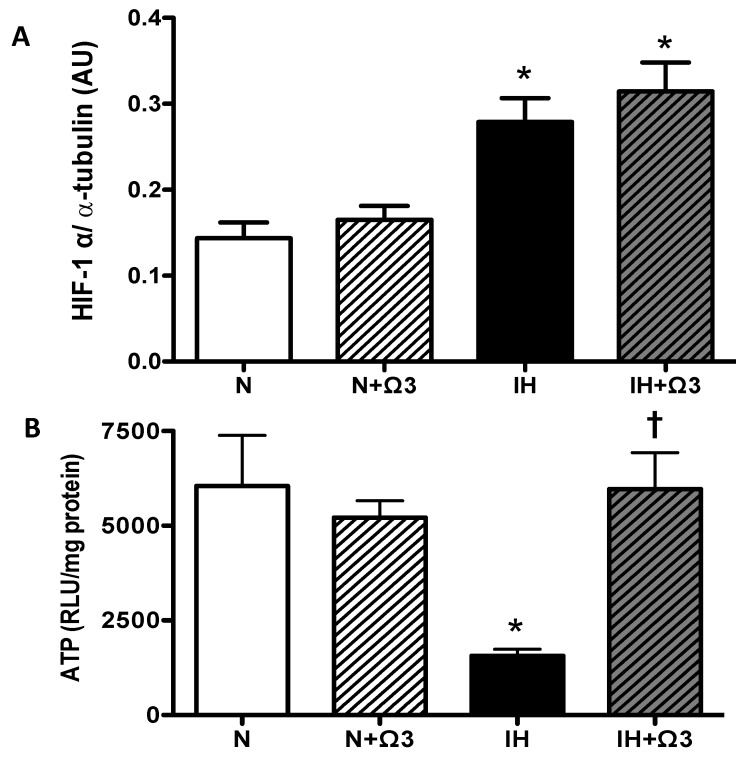
*Hypoxic parameters.* (**A**) *HIF-1α* and (**B**) *ATP* amounts*.* Groups are normobaric normoxic (N), N + omega 3 (N + Ω3), intermittent hypobaric hypoxic (IH) and IH + omega 3 (IH + Ω3). Parameters measured at 120 min of reperfusion in cardiac tissue. Data expressed as mean ± SEM (*n* = 4 per group). Significant differences (*p* < 0.05): * *vs.* N or N + Ω3 (as appropriate); **^†^** *vs.* IH.

As expected, the hypoxic insult increased cardiac levels of HIF-1α. IH and IH + Ω3 rats exhibited higher levels of HIF-1α, relative to N levels (*p* < 0.05). No significant differences were found between IH and IH + Ω3 groups (Figure 3A). Further, IH group shows a marked drop in ATP amounts compared to N group (*p* < 0.05), amounts that were completely recovered by Ω3 supplementation (Figure 3B). 

### 2.5. Oxidative Stress Markers

**Figure 4:**
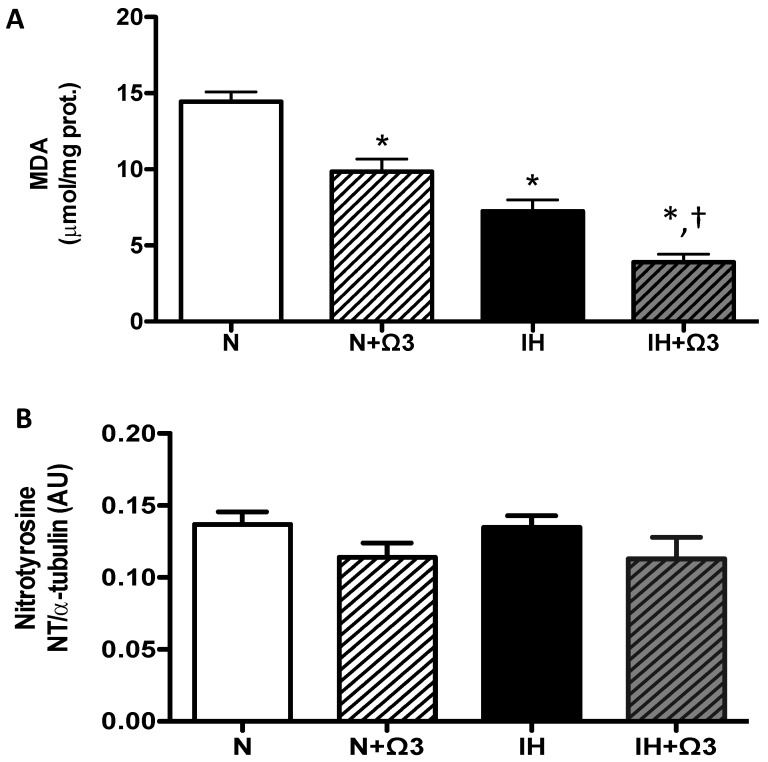
*Oxidative stress markers*. (**A**) MDA and (**B**) Nitrotyrosine levels. Groups are normobaric normoxic (N), N + omega 3 (N + Ω3), intermittent hypobaric hypoxic (IH) and IH + omega 3 (IH + Ω3). Parameters measured at 120 min of reperfusion in cardiac tissue. Data expressed as mean ± SEM (*n* = 7 per group). Significant differences (*p* < 0.05): * *vs.* N; **^†^** *vs.* IH.

Cardiac oxidative stress was assessed by malondialdehyde (MDA) and nitrotyrosine (NT) levels. MDA levels were lower in IH and IH + Ω3 groups, compared to N and IH groups (*p* < 0.05), respectively (Figure 4A). However, NT expression was similar among the four experimental groups (Figure 4B).

### 2.6. Antioxidant Enzymes Levels

**Figure 5:**
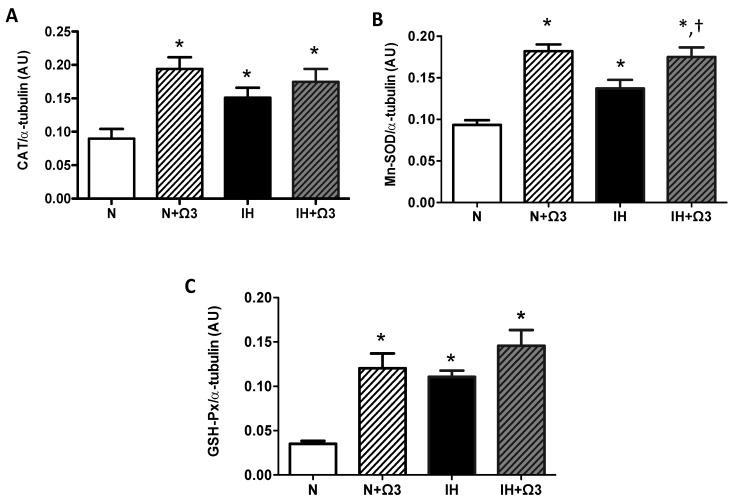
*Antioxidant enzymes expression.* Catalase, CAT (**A**); superoxide dismutase, SOD (**B**) and glutathione peroxidase, GSH-Px (**C**). Groups are normobaric normoxic (N), N + omega 3 (N + Ω3), intermittent hypobaric hypoxic (IH) and IH + omega 3 (IH + Ω3). Parameters measured at 120 min of reperfusion in cardiac tissue. Data expressed as mean ± SEM (*n* = 4 per group). Significant differences (*p* < 0.05): * *vs.* N or N + Ω3 (as appropriate); **^†^** *vs.* IH.

Higher levels of CAT, Mn-SOD and GSH-Px were found in hearts of IH group compared with N group (*p* < 0.05). Further, Ω3 supplementation increased all the antioxidant enzymes expression in normoxia. However, this increase was only observed in Mn-SOD from IH group (*p* < 0.05) (Figure 5B). Thus, no significant differences were found in CAT and GSH-Px levels in IH + Ω3 compared with IH group (Figure 5A–C).

### 2.7. Pro-Inflammatory Markers

**Figure 6:**
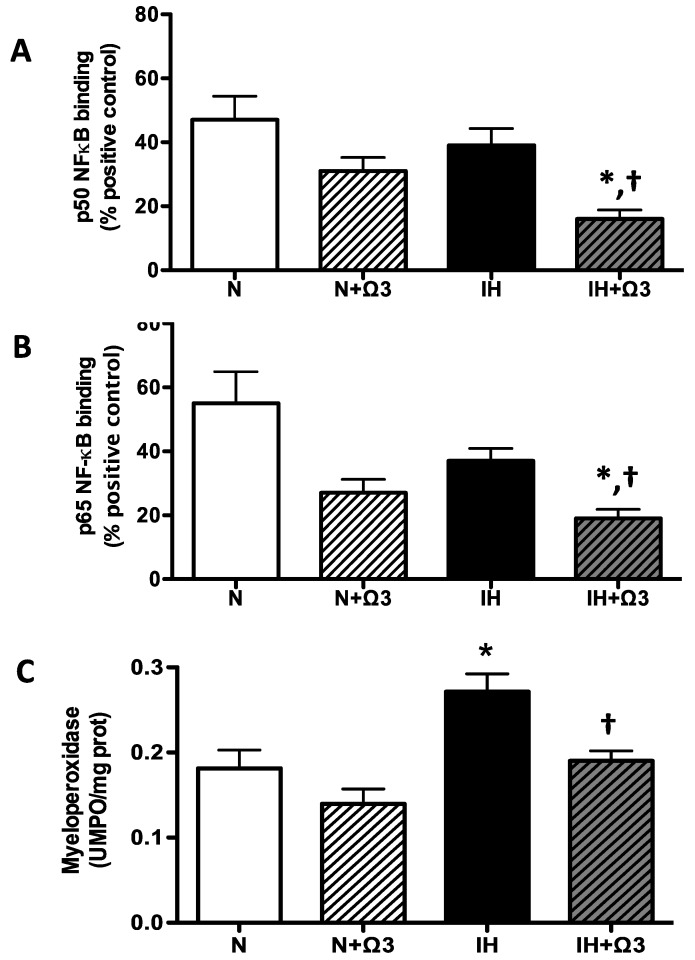
*Pro-inflammatory markers.* p50 (**A**) and p65 NF-κB DNA binding (**B**) and Myeloperoxidase (MPO) activity (**C**). Groups are normobaric normoxic (N), N+omega 3 (N+Ω3), intermittent hypobaric hypoxic (IH) and IH+omega 3 (IH+Ω3). Parameters measured at 120 min of reperfusion cardiac tissue. Data expressed as mean ± SEM. (*n* = 4 per group). Significant differences (*p* < 0.05): * *vs.* N or N**+**Ω3 (as appropriate); **^†^** *vs.* IH.

Cardiac NF-kappaB (NFκB) p50 and p65 subunits DNA binding activity were similar between N and IH groups. However, these variables were lower in IH+Ω3 *vs.* IH rats (*p* < 0.05) (Figure 6A–B). In addition, myeloperoxidase (MPO) in hearts of IH rats was higher compared with N group (*p* < 0.05), effect that was prevented by Ω3 supplementation (*p* < 0.05) (Figure 6C). 

## 3. Discussion

In the present study we showed that both, shifts from hypobaric hypoxia to normobaric normoxia, and Ω3 supplementation, independently exhibited cardioprotective effects in adult rats. Further, we showed that these effects are associated with enhanced antioxidant capacity and decreased oxidative stress in both conditions. However, it seems that there are no synergistic effects between these conditions. Ω3 supplementation induces a pharmacologic preconditioning, limiting myocardial IR injury and endowing cardio-protection in a similar magnitude as IHH preconditioning. 

### 3.1. Cardioprotective Mechanisms Induced by Chronic IHH

It is well known that oxidative stress and reactive oxygen species (ROS) contribute to IR damage [29]. Many proteins that have key roles in the homeostasis of cardiomyocytes, such as the Na^+^/Ca^2+^ exchanger and the sodium-potassium pump, are modified during reperfusion ROS burst [30,31]. Superoxide dismutase is a cytosolic dimeric enzyme, which contributes to the first line of antioxidant defenses of the cell by catalyzing the conversion of superoxide anion (O_2_^−^) into hydrogen peroxide (H_2_O_2_). Both GPX and CAT belong to the secondary line of antioxidant defenses by catalyzing the conversion of H_2_O_2_ to H_2_O. Thus, it is largely believed that the induction of the antioxidant activity in heart tissue may be useful in preventing IR injury [32]. Previous findings have shown that renal SOD levels in rats are increased during hypoxic preconditioning [33]. Cardioprotection by chronic IH has been related to an increase of the antioxidant activity [34,35]. However, the antioxidant enzymes in rats submitted to chronic IH (7000 m altitude, 8 h/day and 5 days/week for 24–30 days) were not significantly altered [36]. In our findings, chronic IH induced preconditioning-like responses together with omega supplementation, improves cardiac recovery after ischemia, through effectively enhancing antioxidant and anti-inflammatory mechanisms (Figure 4 and Figure 5). The cardioprotective nature of chronic IH is affected by several factors, such as the level, duration and protocol of hypoxia [20,23]. For instance, our protocol is consistent with ischemic-like preconditioning and long-term high-altitude hypoxic adaptation, where the cardioprotective effects are reflected by increased tolerance of IR injury, reduced cell death (apoptosis or necrosis), diminished LDH release and improved recovery of cardiac function [18,35,37,38]. These findings are in agreement with our results in which IH groups showed a better recovery of LVDP relative to normoxic ones. Furthermore, the LVEDP data present a low increase at the end of reperfusion, which may reflect a better response to the ventricular hypercontractile phenomenon occurring during the ischemic period. Moreover, it has been reported that the level of IR-induced protein oxidation, assessed by measuring protein carbonylation, following by 1 or 2 weeks of IH, reflects the occurrence of pro-oxidant imbalance in susceptible hearts [39]. In contrast, the hearts from mice exposed to 4 weeks of IH did not have higher levels of oxidative stress, suggesting that the adaptive mechanisms may involve either the reduction of ROS generation, an increased antioxidant capacity, or both [39]. The same pro-oxidant effects were attenuated by the chronic IH in our model, showing lower levels of MDA by the end of the protocol. In the case of antioxidant enzymes activity, the adaptive mechanism could involve the reinforcement of the myocardial antioxidant status. The increases in antioxidant enzymes protein expression may be modulated by hypoxia and/or a raise in ROS [6,23]. Further, the upregulation of redox molecules by transcriptional factors have been previously described for *in vitro* hypoxic models [40,41]. Nuclear factor erythroid 2-related factor 2 (Nrf2) is an essential transcription factor that regulates expression of several antioxidant genes via binding to the antioxidant response element (ARE) and plays a crucial role in cellular defense against oxidative stress [42]. Hypoxic preconditioning can attenuate IR-induced oxidative stress and elicit delayed cardioprotection by upregulating the expression of multiple antioxidant enzymes such as HO-1 and MnSOD, dependent on ARE induction [43,44]. Experimental evidence suggests that hypoxic preconditioning upregulates antioxidant enzymes through activating the Nrf2-ARE pathway and confers delayed cardioprotection, attenuating viability loss and lactate dehydrogenase leakage following IR injury *in vitro* [45]. However, this mechanism of preconditioning induced by chronic hypoxia is incompletely understood, but it is now clear that this delayed cardioprotection of chronic IH against oxidative stress is dependent upon de novo protein synthesis of multiple antioxidant enzymes and pathways [46,47]. 

Some pharmacological strategies against IR injury allow estimating the direct effects on the heart. From this view, the chronic IH effect on the heart is expressed by the induction of cellular response mediated by hypoxic factors, such as HIF-1α, that increased only in the hypoxic groups. HIF-1α is essential for the hypoxic regulation of iNOS gene expression in cardiomyocytes, it is logical to speculate a role for HIF-1α in intermittent hypoxia-induced cardioprotection [48]. Cai *et al.* (2003), showed that in heterozygous HIF-1a-deficient mice (knockout allele at the Hif1a locus), the acute cardioprotection induced by either single or multicycle ischemic preconditioning was absent, suggesting that HIF-1a is necessary for the early window of ischemic preconditioning [49]. The same research group reported that intermittent hypoxia exposure induces HIF-1α-dependent increases in kidney and plasma erythropoietin levels, which leads to the delayed window of cardioprotection in wild type but not in heterozygous HIF-1α-deficient mice [50]. On the other hand, it has been shown that gene silencing of prolyl 4-hydroxylase-2 (PHD2) activates HIF-1α and protects the heart against IR injury [51,52]. Even HIF-1α regulates cardiac metabolism in multiple ways, according to the latter research group, IR-induced substantial inflammatory responses that are characterized by the transcription of proinflammatory chemokines were downregulated by HIF-1α activation. Thereby, the myocardial injury was reduce [52,53]. The inhibition of PHD2 induces protective endoplasmic reticulum stress proteins and attenuates post-ischemic myocardial damage by decreasing the pro-apoptotic components of unfolded protein response [53,54]. Accordingly with this evidence, our results show that intermittent hypobaric hypoxia and PUFA induce the expression of HIF-1α. Indeed, DHA from dietary sources is rapidly incorporated into mitochondrial membranes in adult mice [55]. High DHA levels in phospholipids in mitochondria of eukaryotic cells suggest that DHA-phospholipids are essential for the mitochondrial oxidative phosphorylation (OXPHOS) system, which represents the final biochemical pathway involved in the production of energy in the form of ATP. The effects of the activation of DHA-derived mitochondrial pathways decreased the levels of mitochondrial oxidative stress, reduce mitochondrial c oxidase activity, and increased activities of Mn-SOD [56,57]. 

Since consumption of oxygen is coupled to mitochondrial respiration and ATP production, under hypoxic condition, in which the O_2_ supply is diminished, the production of ATP is dramatically decreased [58]. This energetic condition may be worsened even more by reperfusion. An uncontrolled assembling and opening of the mitochondrial permeability transition pore (mPTP), in the inner mitochondrial membrane, occurs during reperfusion. Thus, the influx of protons from intermembrane space to mitochondrial matrix results in collapse of the mitochondrial inner membrane potential (Ψm), uncoupling of oxidative phosphorylation, and rapid depletion of ATP stores [59]. This effect of ischemia-reperfusion on ATP turnover is in line with our finding regarding the IH-induced drop in ATP levels. Conversely, preconditioning effects, induced by chronic IHH exposure, are associated with decreased opening of MPTP and Ca^2+^ overload [38,53]. These findings support the central role of mitochondrial tolerance against the detrimental effects of IR injury. Noteworthy, we also observed that omega supplementation prevented this effects, which suggests a protective effect on mitochondrial function. In fact, it was reported recently that fish oil containing long chain omega-3 polyunsaturated fatty acids improves mitochondrial function in brains of aged NMRI-mice, by restoring complex I, II and IV efficiencies and oxygen consumption coupled to ATP production [55]. 

### 3.2. Cardioprotective Effects Derived from Ω3 Supplementation

Based on the results from cellular and molecular studies, the cardioprotective effects of supplementation with Ω3 appears to be an association between multiple mechanisms that involve an improvement of cardiac hemodynamic factors, such as blood pressure, left ventricular diastolic filling, heart rate and endothelial function [60]. Recent studies showed that perfusion with Ω3 reduce infarct size and improves hemodynamic parameters in an isolated heart model [61]. In a previous study of our group, we showed that severe IR induced tachyarrhythmias, effect that was attenuated with Ω3 treatment [62]. In contrast, other studies have found no differences in the left ventricular function after IR with PUFA supplementation, despite infarct size reduction [63]. 

The antioxidant effects of Ω3 would be mainly related to its incorporation into the cell membrane and modulation of antioxidant signaling pathways. Fish oil supplementation increases expression and activity of the antioxidant enzyme SOD and decreases TBARS in rats [64]. Oxidized Ω3 fatty acids react directly with the negative regulator of Nrf2, Keap1, initiating Keap1 dissociation with Cullin3, thereby inducing Nrf2-dependent antioxidant gene expression [65]. This Ω3-antioxidant reinforcement is associated with a reduction in the susceptibility of myocytes to ROS-induced IR injury and with an increase in SOD and GSH-Px levels [66]. In our findings, hypoxia *per se* was able to decrease oxidative markers and increase antioxidant enzymes levels. However, Ω3 effects were evidence only in the increased level of SOD, which may be induced by oxidized Ω3 through transcriptional mediators, such as SIRT1 and FOXO [67]. However, we cannot exclude that their activities may be increased in specific subcellular compartments involved in ROS generation. Moreover, EPA and DHA supplementation reduced the urinary F2-isoprostane levels, a marker for oxidative stress, as well as enhanced cellular antioxidant defense systems in humans and rats [68,69].

Ω3 can modulate molecular anti-inflammatory pathways due to direct interaction with membrane proteins, regulating gene expression via nuclear receptors and transcription factors, and conversion of bioactive metabolites [24]. For instance, ischemic cardiac disease is associated with eicosanoid profiles to inflammation-resolving lipid mediators and suppression of acute phase reactant in IR [70]. DHA/EPA derived eicosanoids are less inflammatory than their AA-derived eicosanoid counterparts. Therefore, Ω3 can reduce the production of AA-derived eicosanoids by competing for incorporation into the cell membrane, releasing free AA by PLA2 or by inhibiting the enzyme COX-2 and 5-Lipoxygenase [25,26]. These effects are evidenced by a reduction in neutrophil infiltration, inhibition of NFκB activation, decrease COX-2 expression and derived prostanoid synthesis in animal models of IR [26,71]. These findings are in agreement with our results in which Ω3 may be suppressing neutrophil adhesion and tissue infiltration, resulting in lower levels of MPO in IH+Ω3. Further, Ω3 supplementation inhibits NFκB activation in animal models of IR and in some clinical trials as treatment for cardiovascular diseases [72,73]. The proposed molecular mechanisms include modulation of PPARγ, synthesis of inflammatory lipid mediators such as resolvins and protectins, and novel G-protein coupled receptors that mediate potent anti-inflammatory actions [26]. Moreover, high DHA diet counteracts the reduced SIRT1 that is associated directly with p65 subunit, and deacetylates lys310 residue, both critical for NFκB transcriptional activity [74]. Thus, SIRT1 knockout or knockdown leads to increase NFκB activation and pro-inflammatory cytokine release, while SIRT1 activation inhibits NFκB-induced inflammatory mediators [75]. All these mechanisms support the inhibitory effect of Ω3 on NFκB activation, showed by lower amounts of cardiac p50 and p65 subunits in IH+Ω3 rats. However, the role of NFκB in myocardial ischemia and reperfusion (without Ω3 supplementation) might be dual, with both a cardioprotective role in ischemic preconditioning and a detrimental role during sustained IR [76]. The final established effect may depend on the exposure extension and hypoxic intensity. 

### 3.3. Previous Studies on Chronic IHH

Previous studies have shown that IHH protects the heart against I/R damage, using protocols lasting longer than four weeks [77,78,79,80]. This protection was reflected as an improvement in ventricular function associated with the upregulation of antioxidant enzymes in the heart [81]. Recently, we showed that chronic IHH and Ω3 (hypoxia-normoxia: 4 × 4 days) reduced infarct size, TBARS and IL-1beta levels in rats [82]. These results match with other studies that show an improvement of mitochondrial functionality and a reduced oxidative stress in rats exposed to chronic IHH, probably due to a synergistic effect of IH and other condition [78,80]. Moreover, our results also showed that the body weight was significantly lower in the IHH group than in N group. This effect could be explained by the higher metabolic and ventilatory demands as a consequence of the reduced environmental oxygen [2,83]. Indeed, metabolic effects of chronic IHH are associated with an improved in glucose tolerance and significantly increased VLDL-cholesterol, which can affect long-term cardiovascular function and mortality [84,85]. The present findings imply that chronic IHH could be useful in the clinical setting for the prevention of IR injury in ischemic diseases. Moreover, the antioxidant supplementation in I/R injury may also have beneficial consequences.

## 4. Materials and Methods

### 4.1. Animals

All animal care and procedures were evaluated and approved by the Faculty of Medicine Bioethics Committee (Protocol number: CBA# 0627 FMUCH, Universidad de Chile, Santiago, Chile) and are in accordance with the principles of animal care outlined in the Guide for the Care and Use of Laboratory Animals (Institute of Animal Laboratory Resources, Washington, DC, USA) and the ARRIVE guidelines.

Twenty-eight Wistar male rats (Ten-week-old, initial body weight 211.5 ± 3.4 g) were randomly divided in four equal groups: normobaric normoxia (~750 torr; PO_2_ 156 mmHg; N, *n* = 7); N + Ω3 (*n* = 7), supplemented with PUFA, Acolest TG (720 mg, DHA:EPA = 1.1:1.0; 0.3 g·kg^−^1·d^−1^); hypobaric hypoxia (~428 torr; PO_2_ 90 mmHg; IH, *n* = 7) and, IH + Ω3 (*n* = 7). The hypoxic groups were exposed to 4 cycles of 96 h of hypobaric hypoxia followed by 96 h of normobaric normoxia each, during 32 days. The desired environmental pressure of the hypobaric chamber was achieved by pressure changes simulating altitude increases of 150 m per minute. The animals in the N groups were housed in the same room as the IH at 22 °C in a 12 h/12 h light/darkness. The 4 experimental groups received the same amount of daily food and drinking water (15 g of standard pellet meals; water *ad libitum*). The Ω3 supplementation was maintained during the 32 days of the protocol (normo- or hypo-baric exposure). 

Following the hypobaric or normobaric exposure cycles, rats were anesthetized with pentobarbital (50 mg·kg^−1^ intraperitoneal) for a terminal surgical intervention. Once deep anesthesia was confirmed, a sternotomy was performed and intravenous heparin 100 U·kg^−1^ was administered. Immediately after, venous blood samples were obtained and the heart was promptly excised. 

### 4.2. Ex Vivo Heart—Langendorff Setup

Immediately after dissection, the heart was mounted in a temperature-regulated heart chamber and retrogradely perfused via the ascending aorta using a peristaltic infusion pump (Gilson Minipuls3, F-95400 Villiers-le-Bel, France) at a constant flow of 10–14 mL·min^−1^. This generate an initial mean coronary (aortic) perfusion pressure of 60–70 mmHg with physiological modified Krebs-Henseleit Buffer solution containing (in mM) NaCl (128.3), KCl (4.7), CaCl_2_ (1.35), NaHCO_3_ (20.2), NaH_2_PO_4_ (0.4), MgSO_4_ (1.1), glucose (11.1) and pH 7.4 at 37 °C when equilibrated with a mixture of 95% O_2_/5% CO_2_. Perfusate and bath temperatures were maintained at 37 °C by a thermostatically controlled water circulator (B. Braun Thermomix 1420, Melsungen, Germany). Then, a latex balloon was inserted in the left ventricle through the mitral valve and connected to a pressure transducer (Bridge Amp ML221 AD Instruments, Bella Vista NSW 2153, Australia) and filled with normal saline to produce a left ventricle end-diastolic pressure (LVEDP) of 5–10 mmHg. The volume of the balloon was maintained constant throughout the experiment. The pacing was used to maintain a standard contractile response (300 beats/min). The right atrium was excised to eliminate the contribution of the primary intrinsic pacemaker. The stimulator generated a pacing stimulus of 1-ms duration with an intensity of twice of the threshold current. After 15 min stabilization (basal conditions), hearts with a left ventricular developed pressure (LVDP) less than 60 mmHg and a heart rate (HR) less than 180 bpm were excluded from the study. All hearts were subject to 30 min of global ischemia followed by 120 min reperfusion [62]. 

### 4.3. Left Ventricular Function 

The left ventricle systolic pressure (LVSP), LVEDP and coronary perfusion pressure were measured and continuously recorded throughout the entire experiment on a personal computer using a PowerLab system (ML866 ADInstruments, Bella Vista NSW 2153, Australia). Left ventricular developed pressure (LVDP) was calculated as LVDP = LVSP − LVEDP (mmHg) [86]. 

### 4.4. Energy Consumption 

The composition of the experimental diet (Kcal/Kg) was estimated previously [87]. The total energy consumption was estimated by calculating the caloric value of nutrients, based on experimental diet used. Daily, food intake was estimated by gravimetry [82].

### 4.5. Cellular Injury, Hypoxia, Oxidative Stress and Pro-Inflammatory Markers

#### 4.5.1. Plasmatic Lactate Dehydrogenase

Lactate dehydrogenase (LDH) was measured to evaluate the presence of necrotic cell death. Levels of LDH in plasma were determined fluorometrically according to the manufacturer’s instructions, using a CytoTox-ONE Homogeneous Membrane Integrity Assay kit (Promega, Madison, WI, USA).

#### 4.5.2. Cardiac HIF-1α

Aliquots of tissue homogenate containing equal quantities of proteins, 50 μg, were electrophoresed (100 V) on a 12% SDS/PAGE gel as previously described [88], using primary anti-HIF-1α (sc-10790; 1:250 dilution; Santa Cruz Biotechnology, Santa Cruz, CA, USA), and a secondary anti-rabbit antibody (IC-3R01; 1:1000; Imuny Rheabiotech, SP, Brazil). 

#### 4.5.3. Cardiac ATP

Intracellular ATP levels were quantified by luminescence using a CellTiter-Glo kit from Promega (Madison, WI, USA). Luminescence was measured using a Multi-Mode Microplate Reader (Synergy HT, BioTek, Winooski, VT 05404, USA). Results are expressed as relative luminescence units (RLU) per milligram of protein.

#### 4.5.4. Cardiac Malondialdehyde and Nitrotyrosine

Lipid peroxidation was assessed by the malondialdehyde (MDA) and the amounts of lipid peroxides were detected by high performance liquid chromatography and were expressed as μmol MDA/mg protein [89]. Nitrotyrosine was measured by Western blot with anti-nitrotyrosine (Millipore, 05-233, Darmstadt, Germany) 1/1000 dilution, and a secondary anti-mouse antibody (lot. Number OI192080; 1/5000; Pierce Biotechnology, Rockford, IL, USA). The signals obtained on immunoblot determinations were scanned and quantified by densitometric analysis with a chemoluminescence detection device (Odyssey Imaging System, Li-Cor Biosciences, Lincoln, NE, USA).

#### 4.5.5. Cardiac Antioxidant Enzymes

Protein expression manganese superoxide dismutase (Mn-SOD), catalase (CAT) and glutathione peroxidase (GSH-Px-1) was determined by Western blot with specific antibodies (anti-Mn-SOD, Millipore, 06-984, Darmstadt, Germany 1/1000 dilution; anti-Catalase, Abcam Laboratories, ab1877, Cambridge, UK, 1/10000 dilution and anti-GSH-Px-1, Abcam Laboratories, ab22604, Cambridge, UK, 1/1000 dilution respectively) as described elsewhere [90], the secondary anti-rabbit antibody (IC-3R01; 1:1000; Imuny Rheabiotech, SP, Brazil). The signals obtained on immunoblot determinations were scanned and quantified by densitometric analysis with a chemoluminescence detection device (Odyssey Imaging System, Li-Cor Biosciences, Lincoln, NE, USA).

All protein expression bands obtained by Western blotting were analyzed with Image J Software version 1.46a (NIH, Bethesda, MD, USA) and the integrate density values were normalized by the loading control band (α-tubulin: #3873; 1:500; Cell Signaling, Inc., Danvers, MA, USA). 

#### 4.5.6. Cardiac NF-kappaB Transcription Factor

Nuclear protein extracts from liver samples were prepared with a nuclear extraction kit (Cayman Chemical Company, Ann Arbor, MI, USA), which allows separation of the cytoplasmic and nuclear fractions. Protein concentration in the nuclear fraction was determined with Bradford reagent at 590 nm. A nonradioactive assay kit (Cayman Chemical Company, Ann Arbor, MI, USA) was used for NFκB p50 and p65 DNA binding activities, which evaluates NFκB subunits binding to the response element by ELISA. The results were expressed as percentage of NFκB DNA binding activity relative to a positive control (100%).

#### 4.5.7. Cardiac Myeloperoxidase Activity

Neutrophil infiltration was assessed through the determination of myeloperoxidase activity. Cardiac tissue was homogenized in 50 mM PBS, pH 7.4, and centrifuged at 14,000 *g* for 10 min at 4 °C. The pellet was homogenized again in 50 mM PBS, pH 6.0, containing 0.5% hexadecyltrimethylammonium bromide (HETAB) and 10 mM EDTA. The resulting homogenate was subjected to one cycle of freezing–thawing and a brief period of sonication. An aliquot of homogenate was added to a solution containing 80 mM PBS, pH 5.4, 0.5% HETAB, and 1.6 mM 3,3′,5,5′-tetramethylbenzidine (TMB), and the reaction was started by the addition of 0.3 mM H_2_O_2_. Optical density was read at 655 nm. One unit of myeloperoxidase (UMPO) activity was defined as the amount of enzyme that produced a change in absorbance of 1.0 unit/min at 37 °C [91].

### 4.6. Statistical Analysis

Kolmogorov–Smirnov test was used to check normality. In case that this test resulted in *p* < 0.05, the non-parametric Kruskal–Wallis test was used. The data of the N, N+Ω3, IH and IH+Ω3 were expressed as mean ± SEM, and compared using one-way ANOVA followed by Tukey as a *post-hoc* test, when appropriate (Stata 10.0 for Windows). Differences were considered statistically significant when *p* < 0.05.

## 5. Conclusions

Our findings suggest that both, IH and Ω3 in an independent manner, induce functional improvement by antioxidant and anti-inflammatory mechanisms, establishing cardio-protection. Apparently, the occurrence of these protective mechanisms is determined by temporal exposure of the oxygenation shifts. Permanent exposures to high altitude have been related with increased oxidative stress and cardiovascular failure. Therefore, this study might be describing the initial effects of intermittent hypoxia. The clinical implications of Ω3 supplementation in this model of hypobaric hypoxia could be relevant for occupational health of high altitude exposed workers and high-altitude casual travelers. Further studies are needed for a fully comprehension of the temporal cardiovascular responses to IH. This is relevant for developing public health and labor policies in countries with important populations at highlands. 
